# Cancer patients and coronavirus disease 2019: evidence in context

**DOI:** 10.1186/s12967-020-02483-w

**Published:** 2020-08-15

**Authors:** Maddalena Barba, Eriseld Krasniqi, Gennaro Ciliberto, Patrizia Vici

**Affiliations:** 1 Division of Medical Oncology 2, IRCCS IFO-Regina Elena National Cancer Institute, 00144 Rome, Italy; 2 Scientific Direction, IRCCS IFO-Regina Elena National Cancer Institute, 00144 Rome, Italy

**Keywords:** Cancer patients, COVID-19 pandemic, Health care reorganization, Cancer care

## Abstract

In the rapidly evolving coronavirus disease 2019 (COVID-19) outbreak, inherent literature has been increasing at an impressive rate. Such a dynamic scenario imposes the necessity to define a new framework for cancer care. The first emerging evidence has transmitted contrasting messages with regards to cancer care management. Some authors have hypothesized an increased infection risk for cancer patients, with a more severe disease, requiring a reorganization of health care system that could disrupt an established high quality cancer care routine in many developed countries. Other authors have attempted to interpret data related to cancer patients by better defining their “active status”. We herein present our point of view in the light of current evidence and based on the experience matured at our cancer institute in managing cancer patients during the COVID-19 pandemic. Our core idea is that “active cancer” may be considered a proxy of more recent exposure to diagnostic or therapeutic procedures, and the frequency of access to health care facilities can be predicted as a function of the severity of cancer symptoms. Hence, COVID-19 screening program and the adjustment of cancer care provision in a cancer institutions should be led by this risk model, while awaiting new evidence.

Over the past few months, the outbreak of the coronavirus disease 2019 (COVID-19) has fueled the flourishing of inherent literature at an impressive rate. Similarly to the current pandemic, there are virtually no limits to the broadness and heterogeneity of the attentive audience. In such a rapidly evolving scenario, we feel the imperative need to help define the most appropriate framework in which cancer patients and their needs must be allocated.

A quite alarming message was first conveyed by Liang and colleagues. These authors reported on a case series of 2,007 COVID-19 cases from 575 hospitals throughout China prospectively followed up to Jan 31st 2020. The ratio between the number of COVID-19 patients with cancer and the overall number of COVID-19 patients with available data, i.e., 18/1,590, was compared to the incidence of cancer in the overall China population according to the 2015 report, i.e., 283.83/100,0000 persons/year. The estimates compared were different in nature and the resulting comparison potentially misleading. The authors hypothesized a higher risk of COVID-19 in cancer patients compared to individuals without cancer. Overall, within this COVID-19 case series, detailed data were available for 14 cancer patients only. Among them, four had received chemotherapy or surgery within the last month, while the remaining patients were cancer survivors in routine follow up [1]. The restricted sample size, heterogeneity of the study population, bias and confounding possibly arisen from the study design were discussed elsewhere in reference to the generalizability of this evidence to the entire cancer patients population, at a national and international level [2]. Similarly, extreme caution was recommended concerning the postponement of adjuvant chemotherapy and/or elective surgery for stable cancer in endemic areas, which was suggested by Liang and coauthors. In the adjuvant setting, treatment postponement may significantly worsen patient important outcomes. This latter is a particularly unpleasant and objectionable option when dealing with patients treated with a curative intent. Indeed, we would also discourage from applying a similar reasoning to metastatic patients, particularly in light of the outcomes observed over the past few decades [2, 3]. In addition, beyond the previously cited methodological limitations, the comparison between the Chinese scenario and other countries may be impaired by relevant differences in terms of population demography, modifying effects of non-communicable diseases on SARS-CoV-2 risk of infection and disease, as well as by dissimilarities in the restriction policy issued at a national level [4, 5].

The attempt of characterizing cancer patients by “cancer treatment status” adopted by Liang and colleagues was somewhat recalled by Onder and co-authors, who introduced the term “patients with active cancer”. According to a exquisitely descriptive approach, these authors first presented and discussed data concerning the Italian experience with COVID-19 as to 17th March 2020, with a focus on fatalities. The core message related to an overall fatality rate of about 7.2% (1,625 deaths/22,512 cases with confirmed COVID-19) in Italy, with this rate being higher than that observed in other countries. Population age, definition of COVID-19-related deaths and testing procedures were discussed in reference to the evidence reported. A detailed chart review of 355 deceased Italian patients with COVID-19 showed 72 (20.3%) patients with “active cancer” [6]. This latter and similar reports seem to allocate an “active cancer” status within a 5-year frame. Still, this condition was and subsequently remained not otherwise specified. It is plausible assuming that the performance of procedures related to the diagnostic work up in newly diagnosed and/or recurrent cancer cases, along with therapy administration with either a curative or palliative intent, may all concur to define the activation status of cancer disease. This latter may serve as a filter of reasonable efficacy in performing a first-level screening. Cancer patients may thus ideally fall into the following two major categories: 1. Patients with a recently diagnosed/relapsed cancer, with an indication to further assessment with staging purposes and/or therapeutic decisions and treatment administration, and 2. Patients who are more likely to be on follow up. An “active cancer” may thus be a proxy of more recent exposure to ad hoc diagnostic procedures and/or therapy/ies. Cancer treatments have been optimally discussed elsewhere in reference to their deleterious impact on the immunological status of cancer patients, with potential consequences in terms of increased risk of SARS-Cov-2 infection and less favorable outcomes [2]. The need for a separate discussion concerning the effects of the immune checkpoints inhibitors on patients immunologic profile has also recently emerged [7]. Whichever the therapy administered and/or the specific diagnostic procedure required, in the case of “active cancer”, a high frequency access to the institution of reference is generally required in the short term. Frequent access to healthcare facilities may be associated with higher chances of exposure to the virus, particularly prior to the “stay at home” policy whose strict observance has been not immediately issued. In addition, within such a context, cancer patients with more severe clinical symptoms may have more chances to come to the observation of the healthcare professionals and, consequently, to be considered for testing. Of further relevance, co-morbidities, particularly if multiple, may contribute to affect risk of SARS-CoV-2 infection and development of the inherent disease. Indeed, although limited knowledge is available on the underling mechanisms, particularly unfavorable Covid-19 outcomes have been described in patients with prior pathologic conditions, as well as with high-risk behaviors [6, 8, 9].

As previously stated, the herein cited evidence stemmed from a purely descriptive approach. Our speculation was exclusively inspired by the use of a given terminology, i.e., active cancer, which remains in our view extremely broad and globally inadequate to help define a widely heterogeneous category of patients with extremely varying needs in terms of health assistance. Such a heterogeneity, generally considered worldwide at the national health system level, has now to be opportunely translated to clinical practice under circumstances of emergency.

Results optimization will depend on the health system flexibility and inherent level of information.

Adjunctive data for further analyses and inherent results interpretation are eagerly awaited for a deeper understanding of the actual causes of death in COVID-19 positive deceased patients. Till then, several key questions concerning the optimal management of cancer patients over the current pandemic are likely to remain inadequately framed and the related patients’ needs unmet, with these latter currently including complex and still unclear safety issues. An ideal investigational platform enabling the scientific community to understand and counteract SARS-CoV-2 infection and disease would allow the conduct of adequately sized studies, which, at least in the phase of hypothesis generation, may be observational in nature, although extreme attention should be paid in controlling for confounders and bias. Data on patient-, disease- and virus characteristics should be systematically collected by ad hoc trained research assistants. Future studies should include the collection of biological specimen at pre-defined time points and integrate both old-fashion and innovative, high throughput assessment methodologies. The upcoming trials should encompass both pre-clinical and clinical intervention tasks. Well design observational trials will allow to move quick and informed steps towards the design and conduct of intervention trials. Insights from these studies would apply differently to decision making of health care providers worldwide depending on differences in terms of prevalence and specific courses for the epidemics of interest.

Whichever the specific context, the deriving key elements will inform subsequent steps in health policy programming and conduct of further research tailored on risk of SARS-CoV-2 infection and disease development in specific patients’ categories, including cancer patients.

In the post-pandemic transition, safety needs of both cancer patients and health providers will have to be addressed in parallel with cancer treatment optimization. The new paths to be followed for knowledge generation and transfer will have to be developed and implemented in light of a multidisciplinary approach. This latter will ideally be solidly founded on a multidisciplinary ground and oriented according to a single-patient level focus, highly integrated by the enhancement of the resources available in terms of telemedicine. Particularly in palliative care, telemedicine continues holding the potentials to adequately achieve relevant goals, which span from the management of complex symptoms, including pain, dyspnea, anorexia and delirium, to the provision of qualified emotional support to patients and families, as well as to colleagues of other disciplines. Its multifaceted nature and highly strategic role in the Covid-19 pandemic fully justifies the assertion from Metha and Smith, who state in their recently published viewpoint that “Even in this pandemic, palliative care is not a luxury, it is a necessity” [10].

## Data Availability

Not applicable.
